# Positive Childhood Experiences and Adult Health and Opportunity Outcomes in 4 US States

**DOI:** 10.1001/jamanetworkopen.2025.24435

**Published:** 2025-07-29

**Authors:** Robert D. Sege, Maria V. Aslam, Cora Peterson, Christina Bethell, Dina Burstein, Phyllis Holditch Niolon, Jennifer Jones, Stephanie Ettinger de Cuba, Kelsey Hannan, Elizabeth A. Swedo

**Affiliations:** 1 Institute for Clinical Research and Health Policy Studies, Tufts Medical Center, Boston, Massachusetts; 2 National Center for Injury Prevention and Control, Centers for Disease Control and Prevention, Atlanta, Georgia; 3 Johns Hopkins University Bloomberg School of Public Health, Baltimore, Maryland; 4 Prevent Child Abuse America, Chicago, Illinois; 5 Department of Health Law, Policy & Management, Boston University School of Public Health, Boston, Massachusetts; 6 Department of Pediatrics, Boston University Chobanian & Avedisian School of Medicine, Boston, Massachusetts

## Abstract

**Importance:**

Safe, stable, and nurturing childhood relationships and environments promote optimal child development. It is less understood how these experiences measurably affect individuals’ lifelong health and well-being into adulthood.

**Objective:**

To assess health and life opportunities and economic value associated with positive childhood experiences (PCEs) reported by adults.

**Design, Setting, and Participants:**

This cross-sectional study used Behavioral Risk Factor Surveillance Survey data from adults (≥18 years) from 4 states (Kansas [2020], Montana [2019], South Carolina [2020], and Wisconsin [2015]), analyzed from May 2023 to April 2024.

**Exposure:**

Seven PCEs (adult made you feel safe and protected, felt you belonged in high school, felt supported by friends, at least 2 adults that took an interest in you, felt your family stood by you, enjoyed community traditions, and felt able to talk to your family).

**Main Outcomes and Measures:**

Primary outcomes were PCE prevalence by count (1-2, 3-5, or 6-7 PCEs), adjusted prevalence ratios (aPRs) representing the association of PCEs with adult outcomes, and the estimated economic value of PCE-associated, averted morbidity and mortality for chronic health conditions and risk behaviors. Regression models of adult cohorts by PCE exposure and matched by demographic characteristics were used to assess associations of PCEs with current life opportunities (postsecondary education and income), health risk behaviors (moderate to heavy drinking and smoking), chronic health conditions (arthritis, asthma, cancer, chronic obstructive pulmonary disease [COPD], depression, diabetes, heart disease, kidney disease, overweight or obesity, and stroke), and overall physical and mental health. Prevented fractions for the population were used to calculate the reduced proportion of total disease-specific medical spending and healthy life-years savings associated with PCEs.

**Results:**

The sample size was 20 916 individuals (11 357 female [54.3%]; 4469 aged 55-64 years [21.4%]). Approximately one-half of adults (53.1%; 95% CI, 52.1-54.1) reported a high PCE score. A higher PCE score was associated with increased likelihood of attaining postsecondary education (1-2 PCEs: aPR, 1.23; 95% CI, 1.04-1.06; 3-5 PCEs: aPR 1.44; 95% CI, 1.23-1.67; 6-7 PCEs: aPR, 1.64; 95% CI, 1.42) and greater annual household income (1-2 PCEs: aPR, 1.33; 95% CI, 1.05-1.69; 3-5 PCEs: aPR, 1.74; 95% CI, 1.40-2.16; 6-7 PCEs: aPR, 2.17; 95% CI, 1.75-2.68), and lower likelihood of smoking (3-5 PCEs: aPR, 0.79; 95% CI, 0.70-0.89; 6-7 PCEs: aPR, 0.64; 95% CI, 0.57-0.72). Higher PCE scores were also associated with lower prevalence of most chronic conditions (any condition for those with 3-5 PCEs: aPR, 0.88; 95% CI, 0.83-0.93; any condition for those with 6-7 PCEs: aPR, 0.78; 95% CI, 0.74-0.83) including arthritis, asthma, cancer, COPD, depression, diabetes, heart disease, overweight or obesity, and poor general physical or mental health compared with having no PCEs. The estimated annual economic value of PCE-associated, reduced chronic disease in the 4 analyzed states was $215.9 billion ($3.1 billion in lower medical spending cost and $212.8 billion in additional healthy life-years) for respondents with PCE scores of 3 or more, which corresponded to an estimated $28 132 per adult per year, or $782 000 over their lifetimes.

**Conclusions and Relevance:**

This cross-sectional study quantified the importance of PCEs for lifelong health and well-being. The economic benefits associated with PCEs support consideration of strategies to promote these childhood experiences.

## Key Points

**Question:**

Are adult outcomes associated with exposure to positive childhood experiences (PCEs)?

**Findings:**

In this cross-sectional study of Behavioral Risk Factor Surveillance System data for 20 916 individuals across 4 states collected throughout 2015 to 2020, adults with PCEs had a higher probability of attaining postsecondary education, higher household income, and lower probability of multiple health risk behaviors and chronic diseases. PCEs were associated with an annual economic value of $215.9 billion from reduced disease morbidity and mortality.

**Meaning:**

This cross-sectional study found that PCEs were associated with better life opportunities and reduced health risk behaviors and chronic diseases, supporting the consideration of strategies to promote these childhood experiences.

## Teaser

This cross-sectional study analyzes associations of positive childhood experiences with health and life outcomes in adulthood across 4 US states.

## Introduction

Childhood experiences affect brain development and lifelong health.^1,2,3,4^ Recent research has identified types of positive childhood experiences (PCEs) that are associated with better adult outcomes, including safe, stable, nurturing relationships and environments; feeling a sense of belonging; and having opportunities to live, learn, and play in a safe community.^1,2,5,6,7^ These results complement published associations of adverse childhood experiences (ACEs)—such as experiencing abuse or neglect or growing up with household dysfunction—with worse health and life opportunity outcomes and substantial economic costs.^8,9,10^ PCEs may have the ability to mitigate the negative impacts of ACEs on health.^1,2,3,4^

Despite their potential importance, efforts to surveil PCEs at a population level are relatively nascent. Several existing surveillance systems have recently been enhanced to collect national, state, and local PCE data, and support research on associations of PCEs with health outcomes and risk behaviors. A set of PCE measures constructed using survey items adapted from the Child and Youth Resilience Measure-28^11^ was included as state-added questions on the Centers for Disease Control and Prevention Behavioral Risk Factor Surveillance Survey (BRFSS) in Wisconsin in 2015.^1,11,12,13^ The Wisconsin BRFSS study reported associations of PCEs with lower levels of adult depression and higher levels of adult social and emotional support, even after adjusting for exposure to ACEs.^1^ Other single-state BRFSS studies have shown that adults with high PCEs had lower prevalence of alcohol, tobacco, and other drug use, and improved overall health.^2,5^ In an analysis of 4 states (Kansas, Montana, South Carolina, and Wisconsin), most adults (55%) reported having at least 6 of the 7 assessed PCEs; prevalence varied by race, gender, and sexual orientation but not by state. Higher PCE scores were associated with higher educational and income attainment.^14^ The present study combines BRFSS PCE data from 4 states to assess previously unexamined health outcomes and quantify economic benefits associated with PCEs.

## Methods

This cross-sectional study followed Strengthening the Reporting of Observational Studies in Epidemiology (STROBE) reporting guideline^15^ and applicable elements of the Consolidated Health Economic Evaluation Reporting Standards (CHEERS) reporting guideline.^16^ Combined BRFSS data from Kansas (2020), Montana (2019), South Carolina (2020), and Wisconsin (2015) were used to assess the associations of adults’ self-reported PCEs with current health characteristics and life opportunities. Data included all states with available BRFSS PCE data at the time of the analysis; the year of administration varied by state. This secondary analysis of anonymous public health surveillance data did not constitute human research and therefore did not require institutional review board review or exemption or participant informed consent (Common Rule 45 CFR §46).

BRFSS methods have been reported previously.^17^ Survey response rates by state were 45.0% to 51.5%, and item-level response to PCE questions ranged from 97.3% to 99.6%. Respondents who were not current residents of the survey state (249 respondents [1%]) or who were missing 2 or more PCE items (3728 respondents [15%]) were excluded. Respondents were weighted to represent adults across included states. Using scoring guidelines from the initial formulation and testing of the PCEs measure,^1^ 7 PCE exposures (adult made you feel safe and protected, felt you belonged in high school, felt supported by friends, at least 2 adults that took an interest in you, felt your family stood by you, enjoyed community traditions, and felt able to talk to your family) were classified as affirmative if respondents identified the PCEs as occurring often or most of the time and assessed by count (0, 1-2, 3-5, or 6-7 PCEs).^1,14^ All PCE questions referred to experiences before the age of 18 years. Adult outcomes assessed for association with PCEs included life opportunities (attended or completed college or technical school and annual household income exceeded $50 000), health risk behaviors (moderate or heavy drinking and/or ever smoking ≥100 cigarettes), chronic conditions (arthritis, asthma, cancer, chronic obstructive pulmonary disease [COPD], depression, diabetes, heart disease, kidney disease, overweight or obesity, and stroke), and overall physical and mental health. We included the social constructs of race and ethnicity to better understand inequities in experiences of PCEs and contextualize our findings within prior research concerning disparities in health outcomes by race and ethnicity.^18,19^ Race and ethnicity responses were self-reported using 2 separate questions that were subsequently combined into a calculated race and ethnicity variable with 7 categories: American Indian or Alaskan Native only (non-Hispanic), Asian only (non-Hispanic), Black or African American only (non-Hispanic), Hispanic, multiracial (non-Hispanic), White only (non-Hispanic), and another race only (non-Hispanic; included respondents who reported they are Native Hawaiian, Pacific Islander, or another race group not listed in the question responses and are not of Hispanic origin). All respondents who responded affirmatively to the Hispanic ethnicity question were classified as Hispanic. Study measure definitions are reported in eTable 1 in Supplement 1. We calculated prevalence of PCEs, adjusted prevalence ratios (aPRs) representing the association of PCEs with adult characteristics, and the estimated economic value of PCE-associated lower prevalence of morbidity and mortality for chronic health conditions and risk behaviors. Prevented fractions for the population (PFP) were calculated to examine the reduced burden of chronic disease associated with PCEs, and population attributable fractions (PAFs) were calculated to examine the proportion of adults with improved life opportunities associated with existing prevalence of PCE exposure. PFPs represent the reduced proportion of disease occurrence,^20^ and PAFs represent the increased proportion of life opportunities associated with the existing level of exposure. PFPs and PAFs based on observational data are not definitively causal.^21^ The cost analysis used the societal perspective, including tangible and intangible costs to multiple payers. Annual cost estimates used a 1-year time horizon. Per affected person lifetime estimates were based on annual costs discounted 3% to present value applied to the current average duration of US adult life expectancy (from 18-79 years for all participants). The economic value is presented in terms of total population annual, per affected adult annual, and per affected adult lifetime values.

### Statistical Analysis

Statistical analysis was performed from May 2023 to April 2024 using Stata version 17.0 (StataCorp). Weighted prevalence estimates of PCEs by count are presented for the total population and by sex, state, race and ethnicity, and age group. To better isolate the effect size of PCE exposure in the associations of PCEs and adult characteristics, propensity score matching (psmatch2 package) was used to match persons without PCE exposure to persons with PCEs who had similar demographic characteristics (sex, race and ethnicity, age, and state of residence).^22,23,24^ A one-to-many match used kernel weighting for complex survey designs with a Mahalanobis component to ensure better fit of model covariates.^22,25,26,27^ Propensity score matching resulted in demographic characteristics of adults who did not report PCE exposure closely matching the characteristics of respondents with reported PCE exposures (eTable 2 in Supplement 1), ensuring that any differences in outcomes between persons with or without PCEs exposure were likely associated with PCEs.^28^

Generalized linear models with log link and binomial distribution estimated the association of 1 to 2, 3 to 5, and 6 to 7 PCEs with adult characteristics in terms of aPRs, compared with 0 PCEs as the referent. Following the approach of a recent report^10^ on the economic burden of ACEs-associated chronic disease in adulthood, PCE PFPs were calculated when modeled aPRs indicated a statistically significant association (2-sided *P* < .05) between PCEs and evaluated outcomes. PCE PFPs were calculated as described in Khosravi et al^29^ and multiplied by estimates of total annual medical spending and number of disability-adjusted life-years (a measure of lost life-years owing to ill health, disability, or early death) from the 2019 Global Burden of Disease Study^30,31^ among adults (aged ≥20 years) in the 4 analysis states combined to estimate the reduced proportion of those conditions and risk behaviors statistically associated with PCE exposure. Total US medical spending estimates (public insurance including Medicare, Medicaid, and other government programs; private insurance; or out-of-pocket payments) by health condition or risk behavior were inflated to 2019 US dollars (from original reporting as 2015 US dollars) and apportioned by US state using total medical spending by state of residence from the US Centers for Medicare & Medicaid Services.^30,32,33,34^ Each disability-adjusted life-year (which represents the loss of 1 year of equivalent full health) was valued at $540 000 (2019 USD).^35,36^ This value from the US Department of Health and Human Services is derived from quality-adjusted life expectancy and value of statistical life, a monetary estimate of the collective value placed on mortality risk reduction as derived in research studies through revealed preferences (eg, wage differences for dangerous occupations) or stated preferences from surveys soliciting individual persons’ willingness to pay for mortality risk reduction. Medical spending and disability-adjusted life-year source data were already adjusted for other health issues (eg, medical spending estimate for depression excluded costs for coexisting chronic diseases) and, therefore, consistent with previous similar studies, no other adjustment was applied.

## Results

Our overall sample size was 20 916 individuals (11 357 female [54.3%]; 4469 aged 55-64 years [21.4%]) weighted to represent 9.27 million adults across included states. The weighted sample included 530 American Indian or Alaska Native individuals (2.5%), 129 Asian individuals (0.6%), 1651 Black or African American individuals (7.9%), 567 Hispanic individuals (2.7%), 419 multiracial individuals (2.0%), 17 212 White individuals (82.3%), and 164 individuals who reported another race (0.8%). Overall, approximately one-half of adults (53.1%; 95% CI, 52.1%-54.1%) reported 6 to 7 PCEs, 34.7% (95% CI, 33.7%-35.7%) reported 3 to 5 PCEs, 9.7% (95% CI, 9.1%-10.2%) reported 1 to 2 PCEs, and 2.5% (95% CI, 2.2%-2.9%) reported 0 PCEs (Table 1). Adults in Montana (aPR, 0.90; 95% CI, 0.86-0.94) and Wisconsin (aPR, 0.93; 95% CI, 0.89-0.98) were less likely than adults in Kansas to report 6 to 7 PCEs. Compared with White adults, American Indian and Alaska Native (aPR, 0.70; 95% CI, 0.57-0.86), Black or African American (aPR, 0.89; 95% CI, 0.83-0.96), Hispanic (aPR, 0.73; 95% CI, 0.63-0.84), and multiracial adults (aPR, 0.82; 95% CI, 0.69-0.97), as well as adults who reported another race (aPR, 0.72; 95% CI, 0.53-0.98), were less likely to report 6 to 7 PCEs. Adults aged 35 years or older were more likely to report 6 to 7 PCEs than adults aged 18 to 24 years (35-44 years: aPR, 1.11; 95% CI, 1.01-1.22; 45-54 years: aPR, 1.10; 95% CI, 1.01-1.21; 55-64 years: aPR, 1.10; 95% CI, 1.01-1.20; ≥65 years: aPR, 1.19; 95% CI, 1.10-1.29).

**Table 1.** Prevalence of PCEs by Sociodemographic Characteristics Among US Adults in 4 States, Behavioral Risk Factor Surveillance System, 2015-2020^a^

Measure	All adults No. (%)^b^	0 PCEs (weighted n = 231 750 [95% CI, 203 940-241 020])		1-2 PCEs (weighted n = 899 190 [95% CI, 843 570-954 810])			3-5 PCEs (weighted n = 3 216 690 [95% CI, 3 123 990-3 309 390])		6-7 PCEs (weighted n = 4 922 370 [95% CI, 4 829,670-5 015,070])	
		% (95% CI)	aPR (95% CI)	% (95% CI)	aPR (95% CI)	% (95% CI)		aPR (95% CI)	% (95% CI)	aPR (95% CI)
Overall	20 916 (100)	2.5 (2.2-2.9)	NA	9.7 (9.1-10.3)	NA	34.7 (33.7-35.7)		NA	53.1 (52.1-54.1)	NA
Sex										
Male	9559 (45.7)	2.0 (1.6-2.5)	1 [Reference]	9.2 (8.3-10.1)	1 [Reference]	35.9 (34.4-37.4)		1 [Reference]	53.0 (51.4-54.5)	1 [Reference]
Female	11 357 (54.3)	3.0 (2.6-3.5)	1.55 (1.18-2.04)^c^	10.2 (9.3-11.1)	1.13 (0.99-1.29)	33.6 (32.2-35.0)		0.94 (0.89-1.00)	53.2 (51.8-54.6)	1.00 (0.96-1.04)
State										
Kansas	4456 (21.3)	2.8 (2.2-3.6)	1 [Reference]	8.5 (7.5-9.7)	1 [Reference]	34.0 (32.3-35.9)		1 [Reference]	54.6 (52.7-56.5)	1 [Reference]
Montana	5627 (26.9)	2.6 (2.1-3.2)	0.96 (0.70-1.32)	11.2 (10.1-12.3)	1.36 (1.15-1.59)^c^	35.9 (34.4-37.5)		1.07 (1.00-1.15)^c^	50.3 (48.7-51.9)	0.90 (0.86-0.94)^c^
South Carolina	5950 (28.4)	2.7 (2.1-3.3)	1.00 (0.71-1.40)	9.2 (8.2-10.3)	1.11 (0.93-1.33)	34.6 (32.9-36.3)		1.01 (0.93-1.09)	53.6 (51.9-55.3)	0.98 (0.94-1.03)
Wisconsin	4883 (23.3)	2.2 (1.7-2.8)	0.83 (0.59-1.16)	10.5 (9.4-11.8)	1.27 (1.07-1.52)^c^	35.0 (33.0-36.9)		1.06 (0.98-1.14)	52.3 (50.3-54.3)	0.93 (0.89-0.98)^c^
Race and ethnicity										
Another race^d^	164 (0.8)	4.9 (1.9-12)	2.25 (0.88-5.76)	10.9 (5.3-21.0)	1.19 (0.59-2.39)	45 (32.1-58.6)		1.35 (1.00-1.83)	39.2 (27.8-51.8)	0.72 (0.53-0.98)^c^
American Indian or Alaska Native	530 (2.5)	2.1 (1.1-4.2)	0.90 (0.44-1.82)	14.8 (10.0-21.2)	1.49 (1.02-2.19)^c^	45.4 (37.1-53.9)		1.37 (1.13-1.65)^c^	37.7 (30.3-45.7)	0.70 (0.57-0.86)^c^
Asian	129 (0.6)	2.6 (0.6-10.7)	1.10 (0.25-4.78)	13.4 (6.8-24.5)	1.29 (0.67-2.50)	37.6 (26.8-49.9)		1.10 (0.80-1.51)^c^	46.4 (34.4-58.9)	0.88 (0.67-1.14)^c^
Black or African American	1651 (7.9)	2.2 (1.5-3.4)	0.90 (0.57-1.42)	9.4 (7.4-12.0)	1.02 (0.77-1.34)	39.1 (35.5-42.8)		1.19 (1.07-1.32)	49.2 (45.6-52.9)	0.89d (0.83-0.96)
Hispanic	567 (2.7)	3.2 (1.8-5.7)	1.34 (0.73-2.46)	14.0 (10.8-18.1)	1.42 (1.07-1.88)^c^	43.8 (38.1-49.6)		1.30 (1.13-1.49)^c^	38.9 (33.5-44.6)	0.73 (0.63-0.84)^c^
Multiracial	419 (2.0)	8.1 (4.6-14)	3.57 (1.98-6.44)^c^	10.4 (7.2-14.8)	1.13 (0.78-1.64)	37.6 (30.3-45.4)		1.11 (0.90-1.36)	43.9 (36.7-51.3)	0.82 (0.69-0.97)^d^
White	17 212 (82.3)	2.3 (2.0-2.7)	1 [Reference]	9.3 (8.7-10.0)	1 [Reference]	33.1 (32.1-34.2)		1 [Reference]	55.2 (54.1-56.4)	1 [Reference]
Age, y										
18-24	1259 (6.0)	2.0 (1.2-3.5)	1 [Reference]	10.0 (8.0-12.4)	1 [Reference]	40.3 (36.7-44.0)		1 [Reference]	47.7 (44.0-51.3)	1 [Reference]
25-34	1899 (9.1)	2.6 (1.9-3.6)	1.31 (0.70-2.48)	11.8 (9.9-14.0)	1.18 (0.89-1.55)	39.3 (36.3-42.5)		0.98 (0.87-1.10)	46.3 (43.2-49.4)	0.97 (0.88-1.07)
35-44	2296 (11.0)	3.2 (2.3-4.3)	1.59 (0.85-2.97)	11.4 (9.7-13.4)	1.14 (0.87-1.51)	32.1 (29.4-35.0)		0.80 (0.70-0.90)d	53.3 (50.3-56.2)	1.11 (1.01-1.22)^c^
45-54	2930 (14.0)	3.0 (2.3-4.0)	1.54 (0.82-2.89)	10.6 (9.1-12.3)	1.08 (0.83-1.42)	32.6 (30.3-35.0)		0.83 (0.74-0.93)d	53.8 (51.3-56.3)	1.10 (1.01-1.21)^c^
55-64	4469 (21.4)	2.3 (1.8-2.9)	1.17 (0.63-2.17)	9.0 (7.8-10.3)	0.92 (0.71-1.20)	34.6 (32.6-36.7)		0.88 (0.79-0.98)d	54.2 (52.1-56.3)	1.10 (1.01-1.20)^c^
≥65	8063 (38.5)	2.1 (1.6-2.7)	1.05 (0.57-1.96)	7.0 (6.2-7.9)	0.73 (0.56-0.94)^c^	32.0 (30.5-33.6)		0.83 (0.75-0.92)^c^	58.9 (57.2-60.5)	1.19 (1.10-1.29)^c^

Abbreviations: aPR, adjusted prevalence ratio; NA, Not applicable; PCE, positive childhood experiences.

^a^ Combined Behavioral Risk Factor Surveillance System data from Kansas (2020), Montana (2019), South Carolina (2020), and Wisconsin (2015) were used to assess the association of adults’ self-reported PCEs with current health characteristics and life opportunities. PCEs were classified as affirmative if respondents identified the PCEs occurred often or most of the time. PCE counts were calculated by summing and categorizing affirmative responses (0, 1-2, 3-5, or 6-7 PCEs).

^b^ Weighted population estimate to represent 9.27 million individuals.

^c^ *P* < .05.

^d^ Another race only (non-Hispanic) included respondents who reported they are Native Hawaiian, Pacific Islander, or another race group not listed in the question responses and are not of Hispanic origin.

Compared with adults with 0 PCEs (Table 2), there was progressively higher likelihood of attaining postsecondary education and an income of $50 000 or more among adults with 1 to 2 PCEs (education: aPR, 1.23; 95% CI, 1.04-1.06; income: aPR, 1.33; 95% CI, 1.05-1.69), 3 to 5 PCEs (education: aPR 1.44; 95% CI, 1.23-1.67; income: aPR, 1.74; 95% CI, 1.40-2.16), and 6 to 7 PCEs (education: aPR, 1.64; 95% CI, 1.42-1.90; income: aPR, 2.17; 95% CI, 1.75-2.68). Exposure to 3 to 5 PCEs (aPR, 0.79; 95% CI, 0.70-0.89) and 6 to 7 PCEs (aPR, 0.64; 95% CI, 0.57-0.72) were each associated with progressively lower likelihood of smoking compared with those with 0 PCEs. Higher PCE scores were associated with lower incidence of poor mental health (3-5 PCEs: aPR; 0.70; 95% CI, 0.66-0.90; 6-7 PCEs: aPR, 0.54; 95% CI, 0.47-0.63) or physical health (1-2 PCEs: aPR, 0.73; 95% CI, 0.60-0.89; 3-5 PCEs: aPR, 0.74; 95 CI, 0.62-0.87; 6-7 PCEs: aPR, 0.55; 95% CI, 0.47-0.65). Higher PCE scores were also associated with lower prevalence of most chronic conditions (any condition for those with 3-5 PCEs: aPR, 0.88; 95% CI, 0.83-0.93; any condition for those with 6-7 PCEs: aPR, 0.78; 95% CI, 0.74-0.83), except for stroke and kidney disease. Population estimates of health conditions prevented and the economic effects of these reductions are reported in Table 3 and eTable 3 in Supplement 1.

**Table 2.** PCEs and Health and Life Opportunity Outcomes Among Adults in 4 States, Behavioral Risk Factor Surveillance System, 2015-2020^a^

Measure	0 PCEs, % (95% CI) [weighted n = 206 057 (2.4%)]^b^	1-2 PCEs (weighted n = 816 016 [9.4%])		3-5 PCEs (weighted n = 2 898 519 [33.4%])		6-7 PCEs (weighted n = 4 775 958 [55.0%])	
		% (95% CI)	aPR (95% CI)	% (95% CI)	aPR (95% CI)	% (95% CI)	aPR (95% CI)
Socioeconomic characteristics^c^							
College education	40.3 (34.5-46.4)	49.7 (45.6-53.8)	1.23 (1.04-1.46)^d^	57.5 (55.1-59.9)	1.44 (1.23-1.67)^d^	67.3 (65.7-69.0)	1.64 (1.42-1.90)^d^
Income ≥$50 000	27.5 (21.8-34.1)	35.4 (31.3-39.7)	1.33 (1.05-1.69)^d^	45.6 (43.1-48.2)	1.74 (1.40-2.16)^d^	59.3 (57.5-61.2)	2.17 (1.75-2.68)^d^
Either	50.9 (44.6-57.2)	57.6 (53.3-61.8)	1.14 (0.99-1.32)	66.9 (64.5-69.2)	1.33 (1.17-1.51)^d^	78.1 (76.6-79.5)	1.51 (1.34-1.71)^d^
Health risk behaviors^c^^,^^e^							
Moderate or heavy drinking	24.5 (19.4-30.4)	24.6 (21.3-28.2)	1.00 (0.76-1.30)	27.4 (25.2-29.8)	1.08 (0.86-1.37)	27.4 (25.9-29.0)	1.10 (0.87-1.37)
Smoking	58.1 (51.8-64.1)	55.4 (51.1-59.6)	1.01 (0.89-1.16)	42.9 (40.7-45.3)	0.79 (0.70-0.89)^d^	36.0 (34.4-37.7)	0.64 (0.57-0.72)^d^
Either	64.7 (58.7-70.3)	63.1 (58.7-67.3)	1.01 (0.90-1.13)	54.7 (52.3-57.0)	0.88 (0.79-0.97)^d^	50.6 (48.9-52.4)	0.80 (0.73-0.89)^d^
Chronic conditions^c^^,^^e^^,^^f^							
Arthritis	39.5 (33.8-45.5)	32.6 (28.9-36.6)	0.90 (0.74-1.09)	27.4 (25.3-29.6)	0.80 (0.67-0.94)^d^	23.3 (22.0-24.6)	0.67 (0.57-0.79)^d^
Asthma	22.5 (18.1-27.5)	21.9 (18.5-25.6)	0.95 (0.73-1.25)	18.0 (16.2-20.0)	0.80 (0.63-1.02)	12.4 (11.3-13.6)	0.56 (0.44-0.71)^d^
Cancer	11.4 (8.6-15.1)	6.6 (5.2-8.3)	0.70 (0.48-1.02)	6.9 (6.1-7.8)	0.71 (0.51-0.99)^d^	7.3 (6.7-8.1)	0.66 (0.47-0.93)^d^
Chronic obstructive pulmonary disease	17.2 (13.0-22.4)	12.0 (9.7-14.8)	0.76 (0.54-1.08)	7.1 (6.2-8.2)	0.52 (0.38-0.71)^d^	4.2 (3.6-4.8)	0.31 (0.23-0.43)^d^
Depression	45.3 (39.2-51.6)	41.4 (37.3-45.6)	0.89 (0.75-1.06)	26.9 (24.8-29.1)	0.60 (0.51-0.70)^d^	14.2 (12.9-15.5)	0.32 (0.27-0.38)^d^
Diabetes	14.3 (10.4-19.2)	11.3 (9-14.0)	0.86 (0.59-1.24)	11.2 (9.5-13.1)	0.87 (0.63-1.21)	9.4 (8.5-10.5)	0.73 (0.54-0.98)^d^
Heart disease	10.1 (7.2-13.9)	7.5 (5.8-9.6)	0.88 (0.57-1.34)	5.7 (5.0-6.6)	0.65 (0.45-0.96)^d^	4.9 (4.4-5.5)	0.53 (0.37-0.77)^d^
Kidney disease^g^	4.7 (2.0-10.3)	4.9 (2.2-10.4)	1.17 (0.39-3.52)	3.2 (2.4-4.2)	0.86 (0.39-1.90)	2.3 (1.8-2.8)	0.58 (0.27-1.24)
Stroke	4.5 (3.1-6.5)	3.7 (2.5-5.4)	1.16 (0.66-2.03)	3.6 (2.4-5.3)	1.08 (0.60-1.95)	2.6 (2.1-3.1)	0.73 (0.44-1.22)
Overweight or obesity^h^	72.8 (66.0-78.6)	71.7 (67.1-75.9)	1.00 (0.89-1.13)	68.9 (66.0-71.7)	0.97 (0.87-1.08)	63.2 (60.8-65.5)	0.89 (0.80-1.00)^d^
Any	86.9 (82.2-90.5)	81.1 (77.7-84.2)	0.95 (0.89-1.01)	75.0 (72.9-76.9)	0.88 (0.83-0.93)^d^	66.8 (65.1-68.5)	0.78 (0.74-0.83)^d^
≥2 Conditions	58.0 (51.7-64.1)	51.0 (46.9-55.1)	0.92 (0.80-1.06)	41.1 (38.8-43.5)	0.77 (0.68-0.87)^d^	30.8 (29.4-32.3)	0.57 (0.51-0.65)^d^
General health^c^^,^^e^^,^^f^							
Poor physical health^h^	53.1 (45.8-60.3)	39.1 (34.4-44)	0.73 (0.60-0.89)^d^	37.9 (34.9-40.9)	0.74 (0.62-0.87)^d^	27.5 (25.6-29.4)	0.55 (0.47-0.65)^d^
Poor mental health^h^	57.1 (49.4-64.5)	57.0 (51.7-62.2)	0.97 (0.82-1.14)	44.6 (41.7-47.6)	0.77 (0.66-0.90)^d^	30.6 (28.6-32.7)	0.54 (0.47-0.63)^d^

Abbreviations: aPR, adjusted prevalence ratio; PCE, positive childhood experiences.

^a^ Combined Behavioral Risk Factor Surveillance System data from Kansas (2020), Montana (2019), South Carolina (2020), and Wisconsin (2015). For every outcome, we used propensity score methods to better isolate the exposure effect and to match respondents without PCEs exposure to persons with PCEs on sociodemographic factors unlikely to be affected by the exposure (ie, sex, race and ethnicity, age, and state of residence). PCEs were classified as affirmative if respondents identified the PCEs occurred often or most of the time. PCE counts were calculated by summing and categorizing affirmative responses (0, 1-2, 3-5, or 6-7 PCEs). The reference group was 0 PCEs.

^b^ Weighted population estimate after matching applied.

^c^ Adjusted for location (state) and demographic factors (sex, race and ethnicity, and age).

^d^ *P* < .05.

^e^ Adjusted for life opportunities (college education and income ≥$50 000).

^f^ Adjusted for presence of any health risk behaviors.

^g^ Wisconsin data not included (unavailable).

^h^ South Carolina data not included (unavailable).

**Table 3.** Chronic Disease PFP and Economic Value Associated With PCEs Among Adults in 4 States, Behavioral Risk Factor Surveillance System, 2015-2020^a^

Measure	PFP by PCE count, %			Total burden in the study population						PCE-associated economic value				
										Total population			Per person^b^	
	1-2^c^	3-5^c^	6-7^c^	Any, %^c^	DALY, No.	DALY, $	Medical spending, $	DALY, $	Medical spending, $	Total, $	%	Annual, $		Lifetime, $^d^
Smoking	NA^e^	5.01	7.05	12.05	696 487	376.1 B	88 M	45.3 B	11 M	45.3 B	21	5907		164 000
Arthritis	NA	4.90	6.74	11.64	89 547	48.4 B	3.8 B	5.6 B	442 M	6.1 B	3	791		22 000
Asthma	NA	NA	7.78	7.78	43 060	23.3 B	1.3 B	1.8 B	102 M	1.9 B	1	249		7000
Cancer	NA	7.21	8.80	16.01	784 727	423.8 B	4.4 B	67.8 B	705 M	68.5 B	32	8932		$248 000
Chronic obstructive pulmonary disease	NA	9.82	8.47	18.29	253 803	137.1 B	1.6 B	25.1 B	296 M	25.4 B	12	3304		92 000
Depression	NA	9.47	8.58	18.06	109 788	59.3 B	2.9 B	10.7 B	521 M	11.2 B	5	1463		41 000
Diabetes	NA	NA	5.88	5.88	191 068	103.2 B	5.2 B	6.1 B	303 M	6.4 B	3	830		23 000
Heart disease	NA	7.98	9.10	17.08	458 223	247.4 B	4.3 B	42.3 B	733 M	43.0 B	20	5603		156 000
Overweight or obesity	NA	NA	2.53	2.53	590 236	318.7 B	452 M	8.1 B	11 M	8.1 B	4	1053		29 000
Total	NA	NA	NA	NA	3 216 939	1.7 T	24.0 B	212.8 B	3.1 B	215.9 B	100	28 132		782 000

Abbreviations: B, billion; DALY, disability-adjusted life year; M, million; NA, not applicable; PCEs, positive childhood experience; PFP, prevented fractions for the population; T, trillion.

^a^ Combined Behavioral Risk Factor Surveillance System data from Kansas (2020), Montana (2019), South Carolina (2020), and Wisconsin (2015). PFPs were calculated only for health conditions statistically associated with PCEs (Table 2).^29^ DALYs and medical spending are reported in 2019 USD. PCEs were classified as affirmative if respondents identified the PCEs occurred often or most of the time. PCE counts were calculated by summing and categorizing affirmative responses (0, 1-2, 3-5, or 6-7 PCEs).

^b^ Calculated using number of adults with 3 or more (3-5 or 6-7) PCEs (2 898 519 + 4 775 958 = 7 674 477 individuals) as the denominator.

^c^ Reference group is 0 PCEs.

^d^ Calculated using the PCE per person total annual economic value estimate applied to the number of years from age 18 to 79 years (current US life expectancy) and discounted 3% annually (a standard rate for valuing future health states) to present value.

^e^ PCE categories that were not statistically different from the unexposed (0 PCEs) group were not included in the PFP calculation.

The calculated PFP of COPD associated with any PCEs was 18.29%, suggesting that the burden of COPD was 18.29% lower than it would be if the population had not experienced PCEs. Ranked PCE PFPs for other conditions were 2.53% for overweight or obesity, 5.88% for diabetes, 7.78% for asthma, 11.64% for arthritis, 13.85% for poor mental health, 16.01% for cancer, 17.08% for heart disease, 18.06% for depression, and 24.14% for poor physical health. Overall, the estimated annual economic value of PCE-associated reduced chronic disease implied by these prevented fractions was $215.9 billion in the 4 states—comprising $3.1 billion in lower medical spending cost and $212.8 billion in additional healthy life-years—among adults with 3 to 5 or 6 to 7 PCEs, which corresponded to an estimated $28 132 per adult with PCEs annually and $782 000 over their lifetime. In addition to declines in chronic conditions, PCEs were associated with improved life opportunities, including a PAF of 26.98% for college graduation and 40.66% for income of $50 000 or greater (eTable 3 in Supplement 1).

## Discussion

In this cross-sectional study, higher PCE scores were associated with improvements in selected adult health and life opportunities and reductions in the burden of chronic conditions and risk behaviors. In the 4 study states, the presence of 3 or more PCEs was associated with an annual economic value of $216 billion based on the lower prevalence of chronic diseases and risk behaviors. The magnitude of the associations of PCEs with health outcomes, risk behaviors, and life opportunities—and of PCEs associated with economic value—suggest potential for reducing the burden of chronic conditions and health risk behaviors, and for improving life opportunities through policy. These findings complement prior work on the population effects of preventing ACEs^8,37^ and support policies that promote equitable access to PCEs as well as the prevention of ACEs.

PCEs may promote positive health and life opportunity outcomes through several pathways. One is through direct effects: environments that provide opportunities for exploration, learning, and social interaction lay the foundation for healthy brain development and promising futures.^38^ Early experiences and interventions are associated with positive outcomes^39,40^; PCEs, such as access to high-quality early education, directly support cognitive development and result in better outcomes.^41^ Similarly, PCEs’ promotion of secure attachments, social supports, and nurturing environments provide a strong foundation for self-esteem^42^ and resilience,^43^ which can serve as protective factors against engaging in risk behaviors^2^ and promote positive mental health.^1,44^ Second, PCEs may exert positive effects by preventing ACEs.^45^ Approaches such as early childhood home visitation can help promote nurturing relationships and environments and reduce children’s risk for experiencing violence.^46,47,48^ Well child services are a high leverage opportunity where pediatric primary care clinicians can focus on promoting PCEs and early relational health.^49^ Additionally, prevention efforts that improve community resources and reduce acceptability of violence create spaces where children feel safe and supported in their neighborhood, lessen opportunities for violence, and promote opportunities for community engagement and connection.^48^ Last, decades of research support the importance of positive experiences and relationships in promoting resilience and providing a buffer for children exposed to adversity.^50,51,52^ The study of Wisconsin BRFSS data demonstrated that PCEs can have a large mitigating effect on the association of ACEs with poor mental health, including depression.^1^

Collecting data on PCEs is critical for understanding their associations with important outcomes and creating and evaluating the effectiveness of policies to increase PCEs. This study used a previously validated PCE measure that reflected experiences of relational support and care in family, school, and community settings.^1,2^ Conceptually similar measures have been developed for other applications. The clinically oriented Benevolent Child Experiences scale consists of 10 items measuring recollections of safety and security during childhood.^53^ The National Survey of Children’s Health includes measures about supportive relationships, safe environments, and opportunities for engagement and emotional growth as part of a broader assessment of child health.^7,54,55,56^ Overall, findings from studies examining these measures support the importance of safe, stable, and nurturing relationships and environments that form the core of the Centers for Disease Control and Prevention Essentials for Childhood Framework.^57^ Careful attention to definitions and interpretation must be paid when making comparisons between studies.

Numerous policies exist that support PCEs and promote health, even if not implemented explicitly under a PCE framework. Paid family and medical leave assists families to be able to spend time establishing secure attachments with newborns and to attend to children with complex medical needs, improving family well-being^58^ and reducing risk of child abuse.^59^ Reach Out and Read, a national program that provides children’s books to millions of families, promotes the development of early relational health and has been associated with reducing inequities.^60,61^ Cash support or guaranteed income pilot programs have demonstrated great promise and have been associated with protecting adults’ mental health and sense of self.^62,63,64^ Although each of these examples, and many others, intend to improve family health and increase PCEs, they have differing logic models, measures, and implementation strategies. Adoption of frameworks that explicitly seek to optimize PCEs may facilitate the design, implementation, and evaluation of efforts to improve child and adult health.

Important considerations for such programs are equity and inclusion. Racial-, ethnic-, and nativity-related inequities in material hardships, like housing instability, are well-documented.^65,66,67^ Both material hardships and ACEs have large associations with worse health and well-being across life.^65,66,67,68,69^ Analysis of geocoded data suggests large racial differences in childhood opportunity in the US^70^ that, in turn, reflect root causes of health inequities. Racial and ethnic disparities in PCEs in the dataset used in this study are notable.^14^ Systematic approaches to reducing health inequities by reducing ACEs and promoting PCEs are needed. Careful attention to equity and inclusion in the design of programs and policies can help ensure that all have the opportunity to engage in positive experiences in childhood that can improve their health and life opportunities.

### Limitations

There are several limitations to this study. First, results from 4 states may not be generalizable to the entire US population. The PCE items were offered at the discretion of each state’s public health authority, were not administered during the same year, and the included states were not a random sample of US states. Second, the BRFSS PCE questions do not assess all PCEs that may affect adult outcomes. Unmeasured PCEs may provide additional value that is unaccounted for in our study, and we may be underestimating the associations and cost benefits of PCEs. Third, cross-sectional studies cannot establish causality. Other unmeasured factors, including the effects of systemic racism,^71,72^ may have contributed to the observed associations of PCEs with health outcomes. Fourth, self-reported survey data are subject to possible measurement bias, confounding, recall bias (especially because this module asks adults to recall experiences from childhood), and reporting bias, which might have affected the results. BRFSS responders may differ from nonresponders in demographics and exposure to PCEs. This limitation is likely mitigated, at least in part, by design weighting, which accounts for the probability of selection and adjusts for nonresponse bias and noncoverage errors. Fifth, using an approach similar to a previous study^10^ of the ACE-associated economic burden of chronic disease in the US adult population, this study mathematically combined numerous data inputs to produce point estimates for the economic estimates and did not attempt to combine measures of dispersion to quantify uncertainty in overall measures. Results could underestimate the economic value of PCEs because PCE-associated economic value in childhood was not included. Further, health condition and risk behavior cost data were matched between multiple data sources and may not reflect total costs; for example, medical spending on overweight or obesity was operationalized as the cost of morbid obesity (the best available estimate for data source consistency but likely an overestimate of unit cost). Additionally, Global Burden of Disease data and PFP and related methods are each extensively used but have limitations.^73,74,75^ For these and similar technical reasons, the calculations of economic impact may be imprecise. Sixth, this study did not address ACEs among adults with PCEs. Further research is needed to better understand the roles of both ACEs and PCEs in affecting adult outcomes.

## Conclusions

The results of this cross-sectional study reported large associations of PCEs with adult health outcomes and life opportunities. Policies and initiatives that increase equitable access to these PCEs might improve health outcomes and reduce the economic burden caused by high prevalence of chronic disease and diminished life opportunities. Broader inclusion of PCE questions in state BRFSS surveys could enable more comprehensive research in this area.

## Notes

Supplement 1. **eTable 1.** Study Measure Definitions **eTable 2.** Summary Statistics for Non-Matched as Compared to Matched Behavioral Risk Factor Surveillance System Respondents, 2015-2020 **eTable 3.** Population Attributable Fractions for Life Opportunities and Prevented Fractions for the Population for Health Risk Behaviors and Chronic Conditions Associated With Positive Childhood Experiences, Four States, Behavioral Risk Factor Surveillance System, 2015-2020

Supplement 2.
Data Sharing Statement
