# Supplementary material for: Positive Childhood Experiences and Adult Health and Opportunity Outcomes in 4 US States
Source: JAMA Netw Open. 2025 Jul 29;8(7):e2524435. doi: 10.1001/jamanetworkopen.2025.24435 (PMC12308446; doi:10.1001/jamanetworkopen.2025.24435)
Supplement: Supplement 1. — eTable 1. Study Measure Definitions eTable 2. Summary Statistics for Non-Matched as Compared to Matched Behavioral Risk Factor Surveillance System Respondents, 2015-2020 eTable 3. Population Attributable Fractions for Life Opportunities and Prevented Fractions for the Population for Health Risk Behaviors and Chronic Conditions Associated With Positive Childhood Experiences, Four States, Behavioral Risk Factor Surveillance System, 2015-2020 [file jamanetwopen-e2524435-s001.pdf]

Supplemental Online Content

Sege RD, Aslam MV, Peterson C. Positive childhood experiences and adult health and opportunity outcomes in 4 US states. *JAMA Netw. Open*. 2025;8(7):e2524435. doi:10.1001/jamanetworkopen.2025.24435

eTable 1. Study Measure Definitions

eTable 2. Summary Statistics for Non-Matched as Compared to Matched Behavioral Risk Factor Surveillance System Respondents, 2015-2020

eTable 3. Population Attributable Fractions for Life Opportunities and Prevented Fractions for the Population for Health Risk Behaviors and Chronic Conditions Associated With Positive Childhood Experiences, Four States, Behavioral Risk Factor Surveillance System, 2015-2020

This supplemental material has been provided by the authors to give readers additional information about their work.

© 2025 Sege RD et al. *JAMA Network Open*.

eTable 1. Study Measure Definitions

Measure	Behavioral Risk Factor Surveillance System	Global Burden of Disease Study	Medical spending (Dieleman, 2019)
Exposure: Positive childhood experiences			
PCE: Safety	Adult made you feel safe and protected	NA	NA
PCE: Belonging	Felt you belonged in high school	NA	NA
PCE: Friends	Felt supported by friends	NA	NA
PCE: Adult	At least two adults that took an interest	NA	NA
PCE: Family 1	Felt their family stood by them	NA	NA
PCE: Traditions	Enjoyed community traditions	NA	NA
PCE: Family 2	Felt able to talk to their family	NA	NA
Sociodemographic characteristics			
Sex	Male/female	NA	NA
Race/ethnicity	American Indian or Alaskan Native only (non-Hispanic), Asian only (non-Hispanic), Another race only (non-Hispanic), Black or African American only (non-Hispanic), Hispanic, Multiracial (non-Hispanic, White only (non-Hispanic)	NA	NA
Age, years	18-24, 25-34, 35-44, 45-54, 55-64, and 65+	NA	NA
State	Kansas, Montana, South Carolina, and Wisconsin	NA	NA
Outcomes			
Life opportunities			
College, attended or graduated	Attending or graduating from college or technical school	NA	NA
Income $50,000 or more	Annual household income from all sources	NA	NA
Health risk behaviors			
Moderate or heavy drinking during past 30 days	More than average number of drinks per day during the past 30 days (2 in Wisconsin, 1 in other states)	NA	NA
Smoked 100 cigarettes	Smoked at least 100 cigarettes in lifetime	Smoking	Tobacco intervention
Chronic conditions			
Arthritis	Has a doctor, nurse, or other health professional ever told you that you had some form of arthritis, rheumatoid arthritis, gout, lupus, or fibromyalgia?	Osteoarthritis	Osteoarthritis
Asthma	Has a doctor, nurse, or other health professional ever told you that you had asthma?	Asthma	Asthma
Cancer	Has a doctor, nurse, or other health professional ever told you that you had any other types of cancer [not skin cancer]?	Neoplasms (excluding malignant skin melanoma and nonmelanoma skin cancer)	Neoplasms (excluding malignant skin melanoma and nonmelanoma skin cancer)

© 2025 Sege RD et al. *JAMA Network Open*.

Measure	Behavioral Risk Factor Surveillance System	Global Burden of Disease Study	Medical spending (Dieleman, 2019)
COPD	Has a doctor, nurse, or other health professional ever told you that you had chronic obstructive pulmonary disease, C.O.P.D., emphysema or chronic bronchitis?	Chronic obstructive pulmonary disease	COPD
Depression	Has a doctor, nurse, or other health professional ever told you that you had a depressive disorder (including depression, major depression, dysthymia, or minor depression)?	Depressive disorders	Depressive disorders
Diabetes	Has a doctor, nurse, or other health professional ever told you that you had diabetes?	Diabetes mellitus type 2	Diabetes mellitus
Heart disease	Has a doctor, nurse, or other health professional ever told you that you had a heart attack (myocardial infarction), angina, or coronary heart disease?	Ischemic heart disease plus Hypertensive heart disease	Ischemic heart disease plus Hypertensive heart disease
Kidney disease	Not including kidney stones, bladder infection or incontinence, were you ever told you have kidney disease?	NA	NA
Stroke	Has a doctor, nurse, or other health professional ever told you that you had a stroke?	NA	NA
Overweight or obese	Body Mass Index (BMI) of 25 kg/m^2 or more; yes/no.	High body mass index	Morbid obesity
General health			
Poor physical health	Any number of days within the past 30 days with physical health not good (physical illness or injury)	NA	NA
Poor mental health	Any number of days withing the past 30 days with mental health not good (stress, depression, or emotional problems)	NA	NA

NA indicates variable not available within dataset.

© 2025 Sege RD et al. *JAMA Network Open*.

eTable 2. Summary Statistics for Non-Matched as Compared to Matched Behavioral Risk Factor Surveillance System Respondents, 2015-2020[a]

		PCE exposure, Non-matched			PCE exposure, Matched		
College, attended or graduated		1 (N=20396)	0 (N=471)	Test	1 (N=19805)	0 (N=471)	Test
	Sex						
	Male	(48.7%)	(38.2%)	0.003	(32.3%)	(32.3%)	1.000
	Female	(51.3%)	(61.8%)		(67.7%)	(67.7%)	
	Race/ethnicity						
	African American only, Non-Hispanic	(11.3%)	(10.1%)	0.561	(8.3%)	(8.3%)	1.000
	Hispanic	(5.4%)	(6.8%)	0.459	(4.2%)	(4.2%)	1.000
	Other race/ethnicity categories combined, non-Hispanic	(4.1%)	(7.7%)	0.004	(10.2%)	(10.2%)	1.000
	White only, Non-Hispanic	(79.2%)	(75.5%)	0.189	(77.3%)	(77.3%)	1.000
	Age, years						
	18-34	(27.3%)	(25.3%)	0.540	(15.9%)	(15.9%)	1.000
	35-54	(31.2%)	(38.7%)	0.017	(34.2%)	(34.2%)	1.000
	55+	(41.5%)	(36.0%)	0.076	(49.9%)	(49.9%)	1.000
	State						
	Kansas	(20.7%)	(23.4%)	0.301	(21.0%)	(21.0%)	1.000
	Montana	(7.8%)	(8.0%)	0.765	(25.9%)	(25.9%)	1.000
	South Carolina	(34.8%)	(36.8%)	0.536	(28.2%)	(28.2%)	1.000
	Wisconsin	(36.8%)	(31.8%)	0.129	(24.8%)	(24.8%)	1.000
		PCE exposure, Non-matched			PCE exposure, Matched		
Income $50,000 or more		1 (N=17610)	0 (N=413)	Test	1 (N=17068)	0 (N=413)	Test
	Sex						
	Male	(49.9%)	(40.9%)	0.016	(33.2%)	(33.2%)	1.000
	Female	(50.1%)	(59.1%)		(66.8%)	(66.8%)	
	Race/ethnicity						
	African American only, Non-Hispanic	(10.6%)	(9.5%)	0.611	(7.5%)	(7.5%)	1.000
	Hispanic	(5.3%)	(6.4%)	0.537	(4.4%)	(4.4%)	1.000
	Other race/ethnicity categories combined, non-Hispanic	(4.0%)	(7.7%)	0.003	(10.9%)	(10.9%)	1.000
	White only, Non-Hispanic	(80.1%)	(76.4%)	0.201	(77.2%)	(77.2%)	1.000

© 2025 Sege RD et al. *JAMA Network Open*.

	Age, years						
	18-34	(26.3%)	(25.7%)	0.882	(16.2%)	(16.2%)	1.000
	35-54	(32.9%)	(39.9%)	0.038	(35.6%)	(35.6%)	1.000
	55+	(40.9%)	(34.3%)	0.046	(48.2%)	(48.2%)	1.000
	State						
	Kansas	(20.2%)	(20.2%)	0.976	(18.9%)	(18.9%)	1.000
	Montana	(7.8%)	(8.2%)	0.635	(26.6%)	(26.6%)	1.000
	South Carolina	(33.5%)	(37.1%)	0.300	(27.6%)	(27.6%)	1.000
	Wisconsin	(38.6%)	(34.4%)	0.243	(26.9%)	(26.9%)	1.000
		PCE exposure, Non-matched			PCE exposure, Matched		
College or income $50,000 or more		1 (N=20413)	0 (N=473)	Test	1 (N=19851)	0 (N=473)	Test
	Sex						
	Male	(48.8%)	(38.3%)	0.003	(32.3%)	(32.3%)	1.000
	Female	(51.2%)	(61.7%)		(67.7%)	(67.7%)	
	Race/ethnicity						
	African American only, Non-Hispanic	(11.3%)	(10.0%)	0.544	(8.2%)	(8.2%)	1.000
	Hispanic	(5.4%)	(7.0%)	0.369	(4.4%)	(4.4%)	1.000
	Other race/ethnicity categories combined, non-Hispanic	(4.1%)	(7.6%)	0.004	(10.1%)	(10.1%)	1.000
	White only, Non-Hispanic	(79.2%)	(75.3%)	0.166	(77.2%)	(77.2%)	1.000
	Age, years						
	18-34	(27.3%)	(25.5%)	0.579	(16.1%)	(16.1%)	1.000
	35-54	(31.2%)	(38.5%)	0.020	(34.0%)	(34.0%)	1.000
	55+	(41.5%)	(36.0%)	0.074	(49.9%)	(49.9%)	1.000
	State						
	Kansas	(20.7%)	(23.3%)	0.318	(20.9%)	(20.9%)	1.000
	Montana	(7.8%)	(8.0%)	0.791	(25.8%)	(25.8%)	1.000
	South Carolina	(34.8%)	(37.1%)	0.487	(28.5%)	(28.5%)	1.000
	Wisconsin	(36.8%)	(31.6%)	0.120	(24.7%)	(24.7%)	1.000
		PCE exposure, Non-matched			PCE exposure, Matched		
Moderate or heavy drinking		1 (N=19977)	0 (N=467)	Test	1 (N=19353)	0 (N=467)	Test
	Sex						
	Male	(48.2%)	(36.3%)	<0.001	(31.9%)	(31.9%)	1.000
	Female	(51.8%)	(63.7%)		(68.1%)	(68.1%)	
	Race/ethnicity						
	African American only, Non-Hispanic	(11.1%)	(9.5%)	0.430	(8.1%)	(8.1%)	1.000
	Hispanic	(5.4%)	(7.3%)	0.299	(4.5%)	(4.5%)	1.000
	Other race/ethnicity	(4.1%)	(7.9%)	0.002	(10.3%)	(10.3%)	1.000

© 2025 Sege RD et al. *JAMA Network Open*.

	categories combined, non-Hispanic						
	White only, Non-Hispanic	(79.4%)	(75.3%)	0.138	(77.1%)	(77.1%)	1.000
	Age, years						
	18-34	(27.1%)	(23.6%)	0.254	(15.8%)	(15.8%)	1.000
	35-54	(31.2%)	(39.2%)	0.011	(33.8%)	(33.8%)	1.000
	55+	(41.7%)	(37.2%)	0.148	(50.3%)	(50.3%)	1.000
	State						
	Kansas	(20.8%)	(23.5%)	0.308	(20.8%)	(20.8%)	1.000
	Montana	(7.8%)	(8.3%)	0.622	(26.1%)	(26.1%)	1.000
	South Carolina	(34.0%)	(35.3%)	0.663	(28.1%)	(28.1%)	1.000
	Wisconsin	(37.4%)	(32.9%)	0.172	(25.1%)	(25.1%)	1.000
		PCE exposure, Non-matched			PCE exposure, Matched		
Ever smoked 100 cigarettes in life		1 (N=20064)	0 (N=467)	Test	1 (N=19511)	0 (N=467)	Test
	Sex						
	Male	(48.8%)	(38.1%)	0.002	(32.5%)	(32.5%)	1.000
	Female	(51.2%)	(61.9%)		(67.5%)	(67.5%)	
	Race/ethnicity						
	African American only, Non-Hispanic	(11.4%)	(9.8%)	0.458	(8.1%)	(8.1%)	1.000
	Hispanic	(5.4%)	(7.1%)	0.353	(4.5%)	(4.5%)	1.000
	Other race/ethnicity categories combined, non-Hispanic	(4.1%)	(7.8%)	0.003	(10.3%)	(10.3%)	1.000
	White only, Non-Hispanic	(79.1%)	(75.3%)	0.182	(77.1%)	(77.1%)	1.000
	Age, years						
	18-34	(27.5%)	(25.9%)	0.618	(16.3%)	(16.3%)	1.000
	35-54	(31.4%)	(37.7%)	0.045	(33.6%)	(33.6%)	1.000
	55+	(41.1%)	(36.4%)	0.133	(50.1%)	(50.1%)	1.000
	State						
	Kansas	(19.9%)	(22.6%)	0.288	(20.6%)	(20.6%)	1.000
	Montana	(7.8%)	(8.1%)	0.743	(26.1%)	(26.1%)	1.000
	South Carolina	(35.1%)	(37.1%)	0.536	(28.3%)	(28.3%)	1.000
	Wisconsin	(37.2%)	(32.2%)	0.128	(25.1%)	(25.1%)	1.000
		PCE exposure, Non-matched			PCE exposure, Matched		
Any health risk behaviors		1 (N=20424)	0 (N=473)	Test	1 (N=19511)	0 (N=467)	Test
	Sex						
	Male	(48.8%)	(38.3%)	0.003	(34.2%)	(38.1%)	0.251
	Female	(51.2%)	(61.7%)		(65.8%)	(61.9%)	
	Race/ethnicity						

© 2025 Sege RD et al. *JAMA Network Open*.

	African American only, Non-Hispanic	(11.3%)	(10.0%)	0.544	(10.6%)	(9.8%)	0.695
	Hispanic	(5.4%)	(7.0%)	0.367	(7.4%)	(7.1%)	0.912
	Other race/ethnicity categories combined, non-Hispanic	(4.1%)	(7.6%)	0.004	(7.9%)	(7.8%)	0.941
	White only, Non-Hispanic	(79.2%)	(75.3%)	0.166	(74.1%)	(75.3%)	0.698
	Age, years						
	18-34	(27.3%)	(25.5%)	0.580	(28.2%)	(25.9%)	0.479
	35-53	(31.2%)	(38.5%)	0.019	(38.0%)	(37.7%)	0.919
	55+	(41.5%)	(36.0%)	0.073	(33.7%)	(36.4%)	0.369
	State						
	Kansas	(20.7%)	(23.3%)	0.319	(21.1%)	(22.6%)	0.577
	Montana	(7.8%)	(8.0%)	0.791	(7.5%)	(8.1%)	0.498
	South Carolina	(34.8%)	(37.1%)	0.485	(34.8%)	(37.1%)	0.488
	Wisconsin	(36.8%)	(31.6%)	0.120	(36.6%)	(32.2%)	0.187
		PCE exposure, Non-matched			PCE exposure, Matched		
Arthritis		1 (N=20331)	0 (N=468)	Test	1 (N=19774)	0 (N=468)	Test
	Sex						
	Male	(48.8%)	(38.0%)	0.002	(32.3%)	(32.3%)	1.000
	Female	(51.2%)	(62.0%)		(67.7%)	(67.7%)	
	Race/ethnicity						
	African American only, Non-Hispanic	(11.3%)	(10.1%)	0.583	(8.3%)	(8.3%)	1.000
	Hispanic	(5.4%)	(7.1%)	0.347	(4.5%)	(4.5%)	1.000
	Other race/ethnicity categories combined, non-Hispanic	(4.1%)	(7.5%)	0.006	(9.8%)	(9.8%)	1.000
	White only, Non-Hispanic	(79.2%)	(75.3%)	0.164	(77.4%)	(77.4%)	1.000
	Age, years						
	18-34	(27.3%)	(25.7%)	0.630	(16.2%)	(16.2%)	1.000
	35-54	(31.3%)	(38.9%)	0.016	(34.2%)	(34.2%)	1.000
	55+	(41.5%)	(35.5%)	0.052	(49.6%)	(49.6%)	1.000
	State						
	Kansas	(20.7%)	(23.5%)	0.288	(21.2%)	(21.2%)	1.000
	Montana	(7.7%)	(8.0%)	0.769	(25.6%)	(25.6%)	1.000
	South Carolina	(34.8%)	(37.0%)	0.502	(28.4%)	(28.4%)	1.000
	Wisconsin	(36.8%)	(31.5%)	0.111	(24.8%)	(24.8%)	1.000
		PCE exposure, Non-matched			PCE exposure, Matched		

© 2025 Sege RD et al. *JAMA Network Open*.

Asthma		1 (N=20381)	0 (N=471)	Test	1 (N=19819)	0 (N=471)	Test
	Sex						
	Male	(48.8%)	(38.2%)	0.003	(32.3%)	(32.3%)	1.000
	Female	(51.2%)	(61.8%)		(67.7%)	(67.7%)	
	Race/ethnicity						
	African American only, Non-Hispanic	(11.3%)	(10.1%)	0.567	(8.3%)	(8.3%)	1.000
	Hispanic	(5.4%)	(7.1%)	0.356	(4.5%)	(4.5%)	1.000
	Other race/ethnicity categories combined, non-Hispanic	(4.1%)	(7.7%)	0.004	(10.2%)	(10.2%)	1.000
	White only, Non-Hispanic	(79.2%)	(75.1%)	0.150	(77.1%)	(77.1%)	1.000
	Age, years						
	18-34	(27.3%)	(25.6%)	0.618	(16.1%)	(16.1%)	1.000
	35-54	(31.2%)	(38.8%)	0.016	(34.2%)	(34.2%)	1.000
	55+	(41.5%)	(35.6%)	0.054	(49.7%)	(49.7%)	1.000
	State						
	Kansas	(20.7%)	(23.5%)	0.291	(21.0%)	(21.0%)	1.000
	Montana	(7.7%)	(8.1%)	0.741	(25.9%)	(25.9%)	1.000
	South Carolina	(34.8%)	(37.1%)	0.485	(28.5%)	(28.5%)	1.000
	Wisconsin	(36.8%)	(31.4%)	0.105	(24.6%)	(24.6%)	1.000
		PCE exposure, Non-matched			PCE exposure, Matched		
Cancer		1 (N=20394)	0 (N=470)	Test	1 (N=19836)	0 (N=470)	Test
	Sex						
	Male	(48.8%)	(38.2%)	0.003	(32.1%)	(32.1%)	1.000
	Female	(51.2%)	(61.8%)		(67.9%)	(67.9%)	
	Race/ethnicity						
	African American only, Non-Hispanic	(11.3%)	(10.0%)	0.551	(8.3%)	(8.3%)	1.000
	Hispanic	(5.4%)	(7.0%)	0.355	(4.5%)	(4.5%)	1.000
	Other race/ethnicity categories combined, non-Hispanic	(4.1%)	(7.5%)	0.007	(9.8%)	(9.8%)	1.000
	White only, Non-Hispanic	(79.2%)	(75.5%)	0.182	(77.4%)	(77.4%)	1.000
	Age, years						
	18-34	(27.3%)	(25.5%)	0.590	(16.2%)	(16.2%)	1.000
	35-54	(31.2%)	(38.6%)	0.019	(34.0%)	(34.0%)	1.000
	55+	(41.5%)	(35.9%)	0.069	(49.8%)	(49.8%)	1.000
	State						

© 2025 Sege RD et al. *JAMA Network Open*.

	Kansas	(20.7%)	(23.4%)	0.309	(21.1%)	(21.1%)	1.000
	Montana	(7.7%)	(8.0%)	0.818	(25.5%)	(25.5%)	1.000
	South Carolina	(34.8%)	(37.0%)	0.513	(28.5%)	(28.5%)	1.000
	Wisconsin	(36.8%)	(31.7%)	0.128	(24.9%)	(24.9%)	1.000
		PCE exposure, Non-matched			PCE exposure, Matched		
COPD		1 (N=20340)	0 (N=466)	Test	1 (N=19782)	0 (N=466)	Test
	Sex						
	Male	(48.8%)	(38.4%)	0.003	(32.6%)	(32.6%)	1.000
	Female	(51.2%)	(61.6%)		(67.4%)	(67.4%)	
	Race/ethnicity						
	African American only, Non-Hispanic	(11.3%)	(10.0%)	0.532	(8.2%)	(8.2%)	1.000
	Hispanic	(5.4%)	(7.1%)	0.353	(4.5%)	(4.5%)	1.000
	Other race/ethnicity categories combined, non-Hispanic	(4.1%)	(7.7%)	0.003	(10.3%)	(10.3%)	1.000
	White only, Non-Hispanic	(79.2%)	(75.2%)	0.157	(77.0%)	(77.0%)	1.000
	Age, years						
	18-34	(27.3%)	(25.7%)	0.624	(16.3%)	(16.3%)	1.000
	35-54	(31.2%)	(38.4%)	0.022	(34.1%)	(34.1%)	1.000
	55+	(41.5%)	(35.8%)	0.070	(49.6%)	(49.6%)	1.000
	State						
	Kansas	(20.6%)	(23.2%)	0.339	(20.8%)	(20.8%)	1.000
	Montana	(7.7%)	(8.0%)	0.756	(26.0%)	(26.0%)	1.000
	South Carolina	(34.8%)	(37.1%)	0.485	(28.8%)	(28.8%)	1.000
	Wisconsin	(36.8%)	(31.7%)	0.124	(24.5%)	(24.5%)	1.000
		PCE exposure, Non-matched			PCE exposure, Matched		
Depression		1 (N=20341)	0 (N=470)	Test	1 (N=19782)	0 (N=470)	Test
	Sex						
	Male	(48.8%)	(38.4%)	0.003	(32.3%)	(32.3%)	1.000
	Female	(51.2%)	(61.6%)		(67.7%)	(67.7%)	
	Race/ethnicity						
	African American only, Non-Hispanic	(11.3%)	(9.8%)	0.477	(8.1%)	(8.1%)	1.000
	Hispanic	(5.4%)	(7.1%)	0.346	(4.5%)	(4.5%)	1.000
	Other race/ethnicity categories combined, non-Hispanic	(4.1%)	(7.6%)	0.005	(10.0%)	(10.0%)	1.000

© 2025 Sege RD et al. *JAMA Network Open*.

	White only, Non-Hispanic	(79.2%)	(75.6%)	0.198	(77.4%)	(77.4%)	1.000
	Age, years						
	18-34	(27.2%)	(25.6%)	0.613	(16.2%)	(16.2%)	1.000
	35-54	(31.2%)	(38.6%)	0.018	(34.0%)	(34.0%)	1.000
	55+	(41.5%)	(35.8%)	0.062	(49.8%)	(49.8%)	1.000
	State						
	Kansas	(20.7%)	(23.4%)	0.305	(21.1%)	(21.1%)	1.000
	Montana	(7.7%)	(7.9%)	0.831	(25.7%)	(25.7%)	1.000
	South Carolina	(34.7%)	(36.8%)	0.522	(28.3%)	(28.3%)	1.000
	Wisconsin	(36.8%)	(31.8%)	0.131	(24.9%)	(24.9%)	1.000
		PCE exposure, Non-matched			PCE exposure, Matched		
Diabetes		1 (N=20410)	0 (N=473)	Test	1 (N=19851)	0 (N=473)	Test
	Sex						
	Male	(48.8%)	(38.3%)	0.003	(32.3%)	(32.3%)	1.000
	Female	(51.2%)	(61.7%)		(67.7%)	(67.7%)	
	Race/ethnicity						
	African American only, Non-Hispanic	(11.3%)	(10.0%)	0.546	(8.2%)	(8.2%)	1.000
	Hispanic	(5.4%)	(7.0%)	0.370	(4.4%)	(4.4%)	1.000
	Other race/ethnicity categories combined, non-Hispanic	(4.1%)	(7.6%)	0.004	(10.1%)	(10.1%)	1.000
	White only, Non-Hispanic	(79.2%)	(75.3%)	0.166	(77.2%)	(77.2%)	1.000
	Age, years						
	18-34	(27.3%)	(25.5%)	0.581	(16.1%)	(16.1%)	1.000
	35-54	(31.2%)	(38.5%)	0.019	(34.0%)	(34.0%)	1.000
	55+	(41.5%)	(36.0%)	0.073	(49.9%)	(49.9%)	1.000
	State						
	Kansas	(20.7%)	(23.3%)	0.321	(20.9%)	(20.9%)	1.000
	Montana	(7.8%)	(8.0%)	0.793	(25.8%)	(25.8%)	1.000
	South Carolina	(34.8%)	(37.1%)	0.489	(28.5%)	(28.5%)	1.000
	Wisconsin	(36.8%)	(31.6%)	0.122	(24.7%)	(24.7%)	1.000
		PCE exposure, Non-matched			PCE exposure, Matched		
Heart disease		1 (N=20422)	0 (N=473)	Test	1 (N=19860)	0 (N=473)	Test
	Sex						
	Male	(48.8%)	(38.3%)	0.003	(32.3%)	(32.3%)	1.000
	Female	(51.2%)	(61.7%)		(67.7%)	(67.7%)	
	Race/ethnicity						
	African American only, Non-Hispanic	(11.3%)	(10.0%)	0.546	(8.2%)	(8.2%)	1.000

© 2025 Sege RD et al. *JAMA Network Open*.

	Hispanic	(5.4%)	(7.0%)	0.366	(4.4%)	(4.4%)	1.000
	Other race/ethnicity categories combined, non-Hispanic	(4.1%)	(7.6%)	0.004	(10.1%)	(10.1%)	1.000
	White only, Non-Hispanic	(79.2%)	(75.3%)	0.164	(77.2%)	(77.2%)	1.000
	Age, years						
	18-34	(27.3%)	(25.5%)	0.574	(16.1%)	(16.1%)	1.000
	35-54	(31.2%)	(38.5%)	0.019	(34.0%)	(34.0%)	1.000
	55+	(41.5%)	(36.0%)	0.074	(49.9%)	(49.9%)	1.000
	State						
	Kansas	(20.7%)	(23.3%)	0.322	(20.9%)	(20.9%)	1.000
	Montana	(7.8%)	(8.0%)	0.794	(25.8%)	(25.8%)	1.000
	South Carolina	(34.8%)	(37.1%)	0.492	(28.5%)	(28.5%)	1.000
	Wisconsin	(36.7%)	(31.6%)	0.123	(24.7%)	(24.7%)	1.000
		PCE exposure, Non-matched			PCE exposure, Matched		
Kidney disease		1 (N=15608)	0 (N=354)	Test	1 (N=15204)	0 (N=354)	Test
	Sex						
	Male	(48.5%)	(37.5%)	0.008	(32.5%)	(32.5%)	1.000
	Female	(51.5%)	(62.5%)		(67.5%)	(67.5%)	
	Race/ethnicity						
	African American only, Non-Hispanic	(15.1%)	(13.9%)	0.677	(10.2%)	(10.2%)	1.000
	Hispanic	(6.1%)	(9.6%)	0.127	(5.4%)	(5.4%)	1.000
	Other race/ethnicity categories combined, non-Hispanic	(4.5%)	(7.6%)	0.036	(10.2%)	(10.2%)	1.000
	White only, Non-Hispanic	(74.3%)	(68.8%)	0.132	(74.3%)	(74.3%)	1.000
	Age, years						
	18-34	(27.8%)	(27.3%)	0.899	(16.7%)	(16.7%)	1.000
	35-54	(30.3%)	(39.3%)	0.013	(35.9%)	(35.9%)	1.000
	55+	(41.9%)	(33.4%)	0.016	(47.5%)	(47.5%)	1.000
	State						
	Kansas	(32.7%)	(34.1%)	0.705	(28.0%)	(28.0%)	1.000
	Montana	(12.3%)	(11.6%)	0.645	(33.9%)	(33.9%)	1.000
	South Carolina	(55.0%)	(54.3%)	0.852	(38.1%)	(38.1%)	1.000
	Wisconsin	(0.00%)	(0.00%)		(0.00%)	(0.00%)	
		PCE exposure, Non-matched			PCE exposure, Matched		
Stroke		1 (N=20383)	0 (N=472)	Test	1 (N=19821)	0 (N=472)	Test
	Sex						
	Male	(48.8%)	(38.3%)	0.003	(32.2%)	(32.2%)	1.000

© 2025 Sege RD et al. *JAMA Network Open*.

	Female	(51.2%)	(61.7%)		(67.8%)	(67.8%)	
	Race/ethnicity						
	African American only, Non-Hispanic	(11.3%)	(10.0%)	0.550	(8.3%)	(8.3%)	1.000
	Hispanic	(5.4%)	(7.0%)	0.364	(4.4%)	(4.4%)	1.000
	Other race/ethnicity categories combined, non-Hispanic	(4.1%)	(7.7%)	0.004	(10.2%)	(10.2%)	1.000
	White only, Non-Hispanic	(79.2%)	(75.3%)	0.162	(77.1%)	(77.1%)	1.000
	Age, years						
	18-34	(27.3%)	(25.5%)	0.575	(16.1%)	(16.1%)	1.000
	35-54	(31.2%)	(38.6%)	0.019	(34.1%)	(34.1%)	1.000
	55+	(41.5%)	(36.0%)	0.074	(49.8%)	(49.8%)	1.000
	State						
	Kansas	(20.7%)	(23.3%)	0.319	(21.0%)	(21.0%)	1.000
	Montana	(7.7%)	(7.9%)	0.833	(25.6%)	(25.6%)	1.000
	South Carolina	(34.8%)	(37.1%)	0.477	(28.6%)	(28.6%)	1.000
	Wisconsin	(36.8%)	(31.7%)	0.120	(24.8%)	(24.8%)	1.000
		PCE exposure, Non-matched			PCE exposure, Matched		
Overweight or obese		1 (N=13868)	0 (N=317)	Test	1 (N=13277)	0 (N=317)	Test
	Sex						
	Male	(51.5%)	(38.7%)	0.003	(33.4%)	(33.4%)	1.000
	Female	(48.5%)	(61.3%)		(66.6%)	(66.6%)	
	Race/ethnicity						
	African American only, Non-Hispanic	(4.1%)	(2.5%)	0.260	(2.5%)	(2.5%)	1.000
	Hispanic	(5.6%)	(8.7%)	0.214	(4.7%)	(4.7%)	1.000
	Other race/ethnicity categories combined, non-Hispanic	(4.7%)	(7.6%)	0.102	(9.5%)	(9.5%)	1.000
	White only, Non-Hispanic	(85.7%)	(81.1%)	0.170	(83.3%)	(83.3%)	1.000
	Age, years						
	18-34	(27.4%)	(22.9%)	0.234	(15.8%)	(15.8%)	1.000
	35-54	(31.4%)	(40.2%)	0.029	(34.1%)	(34.1%)	1.000
	55+	(41.2%)	(36.9%)	0.271	(50.2%)	(50.2%)	1.000
	State						
	Kansas	(31.4%)	(35.6%)	0.282	(28.1%)	(28.1%)	1.000
	Montana	(11.9%)	(13.0%)	0.489	(36.6%)	(36.6%)	1.000
	South Carolina	(0.00%)	(0.00%)		(0.00%)	(0.00%)	

© 2025 Sege RD et al. *JAMA Network Open*.

	Wisconsin	(56.7%)	(51.4%)	0.199	(35.3%)	(35.3%)	1.000
		PCE exposure, Non-matched			PCE exposure, Matched		
Any chronic condition		1 (N=20441)	0 (N=473)	Test	1 (N=19879)	0 (N=473)	Test
	Sex						
	Male	(48.8%)	(38.3%)	0.003	(32.3%)	(32.3%)	1.000
	Female	(51.2%)	(61.7%)		(67.7%)	(67.7%)	
	Race/ethnicity						
	African American only, Non-Hispanic	(11.3%)	(10.0%)	0.545	(8.2%)	(8.2%)	1.000
	Hispanic	(5.4%)	(7.0%)	0.367	(4.4%)	(4.4%)	1.000
	Other race/ethnicity categories combined, non-Hispanic	(4.1%)	(7.6%)	0.004	(10.1%)	(10.1%)	1.000
	White only, Non-Hispanic	(79.2%)	(75.3%)	0.165	(77.2%)	(77.2%)	1.000
	Age, years						
	18-34	(27.3%)	(25.5%)	0.577	(16.1%)	(16.1%)	1.000
	35-54	(31.2%)	(38.5%)	0.019	(34.0%)	(34.0%)	1.000
	55+	(41.5%)	(36.0%)	0.073	(49.9%)	(49.9%)	1.000
	State						
	Kansas	(20.7%)	(23.3%)	0.322	(20.9%)	(20.9%)	1.000
	Montana	(7.7%)	(8.0%)	0.788	(25.8%)	(25.8%)	1.000
	South Carolina	(34.8%)	(37.1%)	0.489	(28.5%)	(28.5%)	1.000
	Wisconsin	(36.8%)	(31.6%)	0.122	(24.7%)	(24.7%)	1.000
		PCE exposure, Non-matched			PCE exposure, Matched		
2+ chronic conditions		1 (N=20441)	0 (N=473)	Test	1 (N=19879)	0 (N=473)	Test
	Sex						
	Male	(48.8%)	(38.3%)	0.003	(32.3%)	(32.3%)	1.000
	Female	(51.2%)	(61.7%)		(67.7%)	(67.7%)	
	Race/ethnicity						
	African American only, Non-Hispanic	(11.3%)	(10.0%)	0.545	(8.2%)	(8.2%)	1.000
	Hispanic	(5.4%)	(7.0%)	0.367	(4.4%)	(4.4%)	1.000
	Other race/ethnicity categories combined, non-Hispanic	(4.1%)	(7.6%)	0.004	(10.1%)	(10.1%)	1.000
	White only, Non-Hispanic	(79.2%)	(75.3%)	0.165	(77.2%)	(77.2%)	1.000
	Age, years						
	18-34	(27.3%)	(25.5%)	0.577	(16.1%)	(16.1%)	1.000
	35-54	(31.2%)	(38.5%)	0.019	(34.0%)	(34.0%)	1.000

© 2025 Sege RD et al. *JAMA Network Open*.

	55+	(41.5%)	(36.0%)	0.073	(49.9%)	(49.9%)	1.000
	State						
	Kansas	(20.7%)	(23.3%)	0.322	(20.9%)	(20.9%)	1.000
	Montana	(7.7%)	(8.0%)	0.788	(25.8%)	(25.8%)	1.000
	South Carolina	(34.8%)	(37.1%)	0.489	(28.5%)	(28.5%)	1.000
	Wisconsin	(36.8%)	(31.6%)	0.122	(24.7%)	(24.7%)	1.000
		PCE exposure, Non-matched			PCE exposure, Matched		
Poor physical health		1 (N=14410)	0 (N=332)	Test	1 (N=13892)	0 (N=332)	Test
	Sex						
	Male	(49.6%)	(38.3%)	0.008	(33.1%)	(33.1%)	1.000
	Female	(50.4%)	(61.7%)		(66.9%)	(66.9%)	
	Race/ethnicity						
	African American only, Non-Hispanic	(4.3%)	(2.6%)	0.213	(2.7%)	(2.7%)	1.000
	Hispanic	(5.8%)	(8.4%)	0.306	(4.8%)	(4.8%)	1.000
	Other race/ethnicity categories combined, non-Hispanic	(4.7%)	(7.9%)	0.068	(9.6%)	(9.6%)	1.000
	White only, Non-Hispanic	(85.2%)	(81.2%)	0.209	(82.8%)	(82.8%)	1.000
	Age, years						
	18-34	(27.6%)	(24.8%)	0.453	(16.6%)	(16.6%)	1.000
	35-54	(31.8%)	(39.4%)	0.056	(34.0%)	(34.0%)	1.000
	55+	(40.6%)	(35.8%)	0.212	(49.4%)	(49.4%)	1.000
	State						
	Kansas	(31.4%)	(36.3%)	0.199	(28.6%)	(28.6%)	1.000
	Montana	(11.9%)	(12.9%)	0.522	(36.4%)	(36.4%)	1.000
	South Carolina	(0.00%)	(0.00%)		(0.00%)	(0.00%)	
	Wisconsin	(56.7%)	(50.8%)	0.146	(34.9%)	(34.9%)	1.000
		PCE exposure, Non-matched			PCE exposure, Matched		
Poor mental health		1	0	Test	1	0	Test
	Sex						
	Male	(49.5%)	(38.4%)	0.009	(32.9%)	(32.9%)	1.000
	Female	(50.5%)	(61.6%)		(67.1%)	(67.1%)	
	Race/ethnicity						
	African American only, Non-Hispanic	(4.3%)	(2.6%)	0.205	(2.7%)	(2.7%)	1.000
	Hispanic	(5.8%)	(8.3%)	0.310	(4.6%)	(4.6%)	1.000
	Other race/ethnicity categories combined, non-Hispanic	(4.6%)	(7.9%)	0.061	(9.8%)	(9.8%)	1.000

© 2025 Sege RD et al. *JAMA Network Open*.

	White only, Non-Hispanic	(85.3%)	(81.2%)	0.208	(82.9%)	(82.9%)	1.000
	Age, years						
	18-34	(27.6%)	(24.5%)	0.403	(16.5%)	(16.5%)	1.000
	35-54	(31.6%)	(39.3%)	0.053	(33.8%)	(33.8%)	1.000
	55+	(40.8%)	(36.3%)	0.237	(49.7%)	(49.7%)	1.000
	State						
	Kansas	(31.5%)	(36.4%)	0.201	(29.0%)	(29.0%)	1.000
	Montana	(11.9%)	(12.6%)	0.622	(35.7%)	(35.7%)	1.000
	South Carolina	(0.00%)	(0.00%)		(0.00%)	(0.00%)	
	Wisconsin	(56.6%)	(50.9%)	0.163	(35.4%)	(35.4%)	1.000

[a] Combined Behavioral Risk Factor Surveillance System data from Kansas (2020), Montana (2019), South Carolina (2020), and Wisconsin (2015).

© 2025 Sege RD et al. *JAMA Network Open*.

eTable 3. Population Attributable Fractions for Life Opportunities and Prevented Fractions for the Population for Health Risk Behaviors and Chronic Conditions Associated With Positive Childhood Experiences, Four States, Behavioral Risk Factor Surveillance System, 2015-2020[a]

	Positive Childhood Experiences (PCEs)[b]			
**Outcomes**	**1-2 PCEs**	**3-5 PCEs**	**6-7 PCEs**	**Any**
Population attributable fractions (PAFs)				
**Life opportunities[c]**				
College, attended or graduated	3.89	8.51	14.59	26.98
Income $50,000 or more	4.06	12.07	24.53	40.66
**Prevented fractions for the population (PFPs)**				
**Health risk behaviors[c,d]**				
Moderate or heavy drinking, during past 30 days	NA[h]	NA	NA	NA
Ever smoked 100 cigarettes in life	NA	5.01	7.05	12.05
**Chronic health conditions[c,d,e]**				
Arthritis	NA	4.90	6.74	11.64
Asthma	NA	NA	7.78	7.78
Cancer	NA	7.21	8.80	16.01
COPD	NA	9.82	8.47	18.29
Depression	NA	9.47	8.58	18.06
Diabetes	NA	NA	5.88	5.88
Heart disease	NA	7.98	9.10	17.08
Kidney disease[f]	NA	NA	NA	NA
Stroke	NA	NA	NA	NA
Overweight or obese[g]	NA	NA	2.53	2.53
**General health[c,d,e]**				
Poor physical health[g]	7.80	7.32	9.02	24.14
Poor mental health[g]	NA	5.91	7.94	13.85

Abbreviations: CI = confidence interval; COPD = chronic obstructive pulmonary disease; PCE = positive childhood experiences.

[a] Combined Behavioral Risk Factor Surveillance System data from Kansas (2020), Montana (2019), South Carolina (2020), and Wisconsin (2015). All data are survey-weighted.

[b] Comparison group is 0 PCEs.

[c] Adjusted for location (state) and demographic factors (sex, race/ethnicity, and age).

[d] Adjusted for life opportunities (college education or income $50,000+).

[e] Adjusted for presence of any health risk behaviors.

[f] Wisconsin data not included (outcome unavailable).

[g] South Carolina data not included (outcome unavailable).

[h] Positive childhood experience categories that were not statistically different from the unexposed (zero positive childhood experiences) group were not included in the PFP calculation and are indicated by a dash.

© 2025 Sege RD et al. *JAMA Network Open*.

For every outcome (e.g., poor mental health), we used propensity score methods to better isolate the exposure effect and to match respondents without PCEs exposure to persons with PCEs on sociodemographic factors unlikely affected by the exposure (i.e., sex, race/ethnicity, age, and state of residence).

The proportion in each outcome that varies in the population due to PCE exposure is captured either by the population attributable fractions (PAFs) or by the prevented fractions for the population (PFPs). Specifically, PAFs capture the estimated proportion of adults in the four states who experience improved life opportunities due to the existing prevalence of PCE exposure. By contrast, PFPs capture the proportion of chronic conditions or health risk behaviors that declined in the four states due to the current PCE levels. For each outcome, PCE PAFs were calculated with Miettinen's formula, while PFPs were calculated from PAFs as described in Khosravi et al. All PAFs and PFPs were reported only when modeled adjusted prevalence ratios indicated a statistically significant ($p<0.05$) association between PCEs and evaluated outcomes. For all other outcomes, PAFs/PFPs were not reported (NS).

© 2025 Sege RD et al. *JAMA Network Open*.
