# Supplementary material for: Positive Childhood Experiences and Adult Health and Opportunity Outcomes in 4 US States
Source: JAMA Netw Open. 2025 Jul 29;8(7):e2524435. doi: 10.1001/jamanetworkopen.2025.24435 (PMC12308446; doi:10.1001/jamanetworkopen.2025.24435)
Supplement: Supplement 2. — Data Sharing Statement [file jamanetwopen-e2524435-s002.pdf]

Data Sharing Statement

Sege. Positive Childhood Experiences and Adult Health and Opportunity Outcomes in 4 US States. *JAMA Netw Open*. Published July 29, 2025. doi:10.1001/jamanetworkopen.2025.24435

Data
Data available: No

Additional Information
Explanation for why data not available: This study was made possible through data use agreements with 4 different states. Data dictionary and code can be shared with any interested researchers. Parties interested in the data will be provided the contact information for the data leads at the individual states, as the data do not belong to CDC.
